# Benchmark of Deep Encoder-Decoder Architectures for Head and Neck Tumor Segmentation in Magnetic Resonance Images: Contribution to the HNTSMRG Challenge

**DOI:** 10.1007/978-3-031-83274-1_15

**Published:** 2025-03-03

**Authors:** Marek Wodzinski

**Affiliations:** 1 Department of Measurement and Electronics, AGH University of Krakow, Krakow, Poland; 2 Information Systems Institute, University of Applied Sciences Western Switzerland (HES-SO Valais-Wallis), Sierre, Switzerland

**Keywords:** Head and Neck Cancer, Deep Learning, Image Segmentation, HNTSMRG, Challenge, Benchmark, MRI

## Abstract

Radiation therapy is one of the most frequently applied cancer treatments worldwide, especially in the context of head and neck cancer. Today, MRI-guided radiation therapy planning is becoming increasingly popular due to good soft tissue contrast, lack of radiation dose delivered to the patient, and the capability of performing functional imaging. However, MRI-guided radiation therapy requires segmenting of the cancer both before and during radiation therapy. So far, the segmentation was often performed manually by experienced radiologists, however, recent advances in deep learning-based segmentation suggest that it may be possible to perform the segmentation automatically. Nevertheless, the task is arguably more difficult when using MRI compared to e.g. PET-CT because even manual segmentation of head and neck cancer in MRI volumes is challenging and time-consuming. The importance of the problem motivated the researchers to organize the HNTSMRG challenge with the aim of developing the most accurate segmentation methods, both before and during MRI-guided radiation therapy. In this work, we benchmark several different state-of-the-art segmentation architectures to verify whether the recent advances in deep encoder-decoder architectures are impactful for low data regimes and low-contrast tasks like segmenting head and neck cancer in magnetic resonance images. We show that for such cases the traditional residual UNetbased method outperforms (DSC = 0.775/0.701) recent advances such as UNETR (DSC = .617/0.657), SwinUNETR (DSC = 0.757/0.700), or SegMamba (DSC = 0.708/0.683). The proposed method (lWM team) achieved a mean aggregated Dice score on the closed test set at the level of 0.771 and 0.707 for the pre- and mid-therapy segmentation tasks, scoring 14th and 6th place, respectively. The results suggest that proper data preparation, objective function, and preprocessing are more influential for the segmentation of head and neck cancer than deep network architecture.

## 1 Introduction

Radiation therapy is one of the most frequently applied cancer treatments worldwide, especially in the context of head and neck (HN) cancer. Today, radiation therapy (RT) planning using magnetic resonance images (MRI) is becoming increasingly popular, thanks to good soft tissue contrast, lack of radiation dose delivered to the patient, and the availability of functional imaging [15].

However, RT planning requires segmenting the cancer before the therapy (preRT) with potential adaptations requiring updates to the segmentation mask during the therapy (midRT). Manual segmentation is time-consuming and varies strongly between observers, depending on their experience [18]. The task is especially difficult for MR volumes compared to e.g. PET-CT volumes providing immediate tumor metabolic information that usually makes the segmentation easier. The importance of HN tumor segmentation motivated researchers to organize several scientific challenges, e.g. HECKTOR dedicated to the tumor segmentation in PET-CT volumes [1], HaN-Seg combining both CT and MRI modalities in the context of segmenting the organs-at-risk [16,17], SegRap dedicated to segmenting both gross tumor volume and organs-at-risk for radiotherapy planning [13], and now the HNTSMRG challenge dedicated to perform the segmentation using only T2w MR volumes.

The progress in the automatic segmentation of medical volumes is tremendous. Probably the most recognized segmentation framework is nnUNet [11], successfully applied to numerous scientific challenges [5, 6]. Nevertheless, research in this domain persists, yielding an increasing number of segmentation architectures and frameworks. Today, the novel contributions are mainly based on transformer architecture, such as UNETR [9], SwinUNETR [8], SegMamba [21], or foundation models like MedSAM [14]. The transformer architecture avoids the inductive bias present in convolutional networks and enables the network to model long-range relations [12], however, at the cost of larger datasets required for training.

All challenges related to HN cancer segmentation (and the majority of segmentation tasks in medical imaging) suffer from a common limitation associated with the amount of available and annotated data [6]. The number of annotated cases for HN cancer is relatively large when compared to other medical datasets (e.g., HECKTOR - 524 training cases, HNTSMRG - 150 training cases, Top-Cow - 90 training cases [22], SEG.A - 56 training cases, SPPIN - 34 training cases), however it is extremely small when compared to the datasets available in computer vision, often exceeding millions of cases.

An open question arises: Are all the advances in medical image segmentation architectures useful in low data regime tasks like the HN cancer segmentation? Do transformer-based networks improve over the baseline based on UNet? We attempt to answer the question by proposing a contribution to the HNTSMRG challenge. The goal of the HNTSMRG challenge is to propose automatic algorithms that correctly segment the primary gross tumor volumes (GTVp) and the metastatic lymph nodes (GTVn). The challenge is divided into two subtasks. The first one (preRT) is dedicated to segmenting the GTVp and GTVn in T2-weighted volumes acquired before the treatment. The second one (midRT) aims to achieve the same goal, however, during the RT, resulting in a considerably more difficult challenge.

### Contribution:

In this work, we benchmark several encoder-decoder architectures dedicated to the segmentation of 3-D medical volumes. We compare traditional Resiudal UNet (RUNET) to more recent architectures: UNETR, SwinUNETR, and SegMamba. We show that the use of more recent and advanced transformer-based architectures is not beneficial for the HN tumor segmentation. The proposed segmentation architecture achieved a considerably good score, however, not at the level of the best-performing submissions based on the nnUNet framework and its pre- and post-processing capabilities. Importantly, the goal of the contribution is not to propose any novel segmentation method, but to verify the influence of the currently existing building blocks.

## 2 Methods

### 2.1 Dataset

The training dataset consisted of 150 T2-weighted (T2w) sequences of the head and neck region acquired at the MD Anderson Cancer Center. For the preRT task, only the MR volume used for therapy planning was available, while for the midRT task, it was possible to use both the midRT volume and the preRT data registered to the midRT MR volume.

The test set consisted of 50 T2w sequences, sharing similar properties to the training test set. However, the test cases were not released to the participants and the evaluation was performed using the Grand-Challenge (GC) platform.

The ground-truth represented the primary gross tumor volumes (GTVp) and metastatic lymph nodes (GTVn). All the GTVp and GTVn were independently segmented by multiple experts (from 3 to 4 for each volume) and then combined. All observers were medical doctors with at least 2 years of experience in HN tumor segmentation. Finally, the quality of the segmentation was verified by experienced radiologists with more than 10 years of experience and the segmentations were combined using the STAPLE algorithm [19] to provide a single segmentation per patient.

### 2.2 Segmentation Overview

**Fig. 1.**
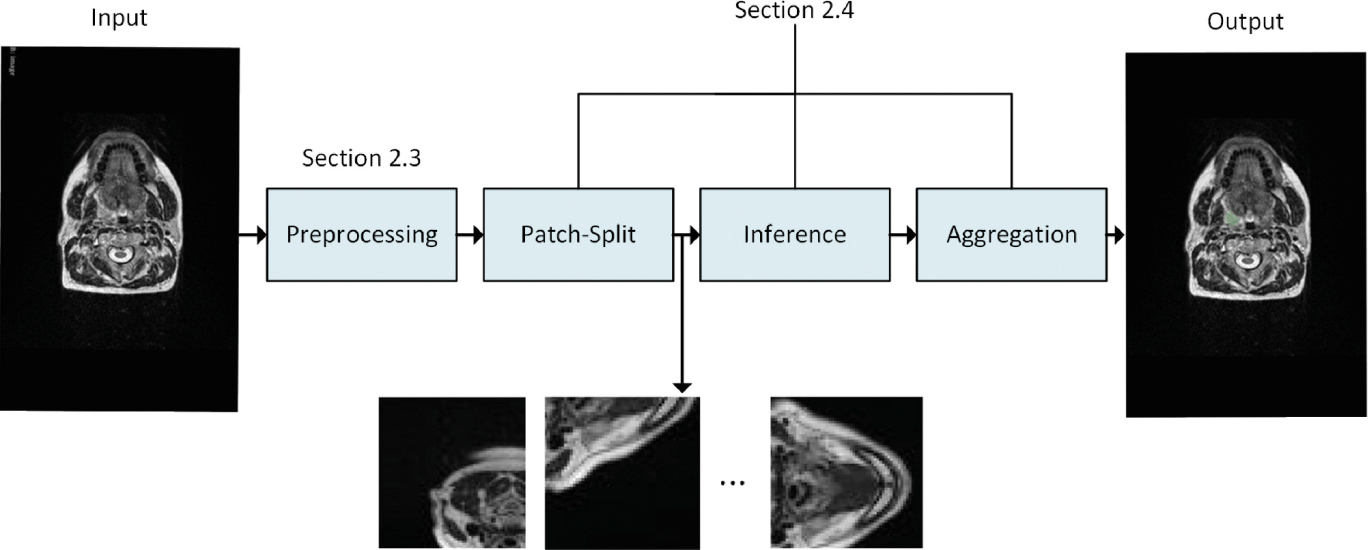
Visualization of the processing pipeline.

The proposed method consisted of (i) preprocessing, (ii) splitting the volume into overlapping patches, (iii) inference using encoder-decoder architecture, and (iv) aggregation of results. The pipeline is presented in Fig. 1.

### 2.3 Preprocessing

The preprocessing was similar for both the preRT and midRT subtasks. It started with resampling all the volumes to isotropic spacing equal to 0.5 mm in each dimension. Then, the pre- and mid-therapy volumes were normalized using the Z-normalization, separately to both volumes.

### 2.4 Inference

The proposed method was patch-based, which means that the input volume was divided into a given number of overlapping patches, the inference was performed separately for each patch, and then the results were aggregated. The patch-based approach was used for two reasons: (i) the limited amount of VRAM that made it impossible to process the volumes directly, (ii) it can be considered as a natural form of augmentation enforcing the network to be able to be more resistant to false positives.

The patch size for all experiments was equal to 128^3^ voxels, allowing the network to use a relatively large region of interest while maintaining computational efficiency. The overlap between patches was set to 32^3^ voxels. The output for each class was calculated by applying the argmax operator to the channel dimension after aggregating the activations from each patch. Due to the heterogeneity of the segmentation masks and the evaluation metric based on the aggregated Dice score, no additional post-processing was applied.

During internal evaluation, only a single model was used for each fold. During the final evaluation using the GC platform, activation maps were aggregated from models trained using each training fold.

### 2.5 Training

The training was implemented using the Lightning [7] and MONAI [3] libraries. The objective function was a weighted combination of SoftDice and Focal losses. The AdamW was used as the optimizer (initial learning rate = 0.001, weight decay = 0.005), and the learning rate was automatically reduced on a plateau by a factor of 0.9 if the validation loss had not improved for more than 10 epochs. The training was accelerated using automatic mixed precision. The training patches were randomly selected from each volume by cropping to each available class (background, GTVn, GTVp) with an equal probability.

### 2.6 Experimental Setup

Two separate networks were trained for Task 1 and Task 2: (i) a single-channel preRT network with the preRT volume as input for Task 1 and (ii) a three-channel midRT network using the concatenated midRT volume, the registered preRT volume, and the associated registered preRT ground truth, for Task 2. All experiments were trained until convergence. The MR volumes (both midRT and preRT) were augmented by random axis flipping, Gaussian and Rician noise, and Gaussian smoothing. The batch size was set to 16, resulting in batches containing 4 random patches from 4 randomly selected volumes. Network architectures, augmentation methods, objective functions, and training utilities were taken from the MONAI library [3]. All experiments were performed using 5-fold cross-validation where each fold consisted of 120 training and 30 validation cases. Any claim about a statistical improvement was supported by a Wilcoxon signed-rank test with a p-value below 0.05.

The training was performed using the PLGRID Helios supercomputing infrastructure with nodes containing 4× NVIDIA GH200 accelerators (4×96 GB VRAM) with automatic mixed-precision enabled. All volumes were initially transferred to the RAM, allowing one to perform fast and efficient training. The training and inference scripts are available in the associated repository [20].

## 3 Results

**Fig. 2.**
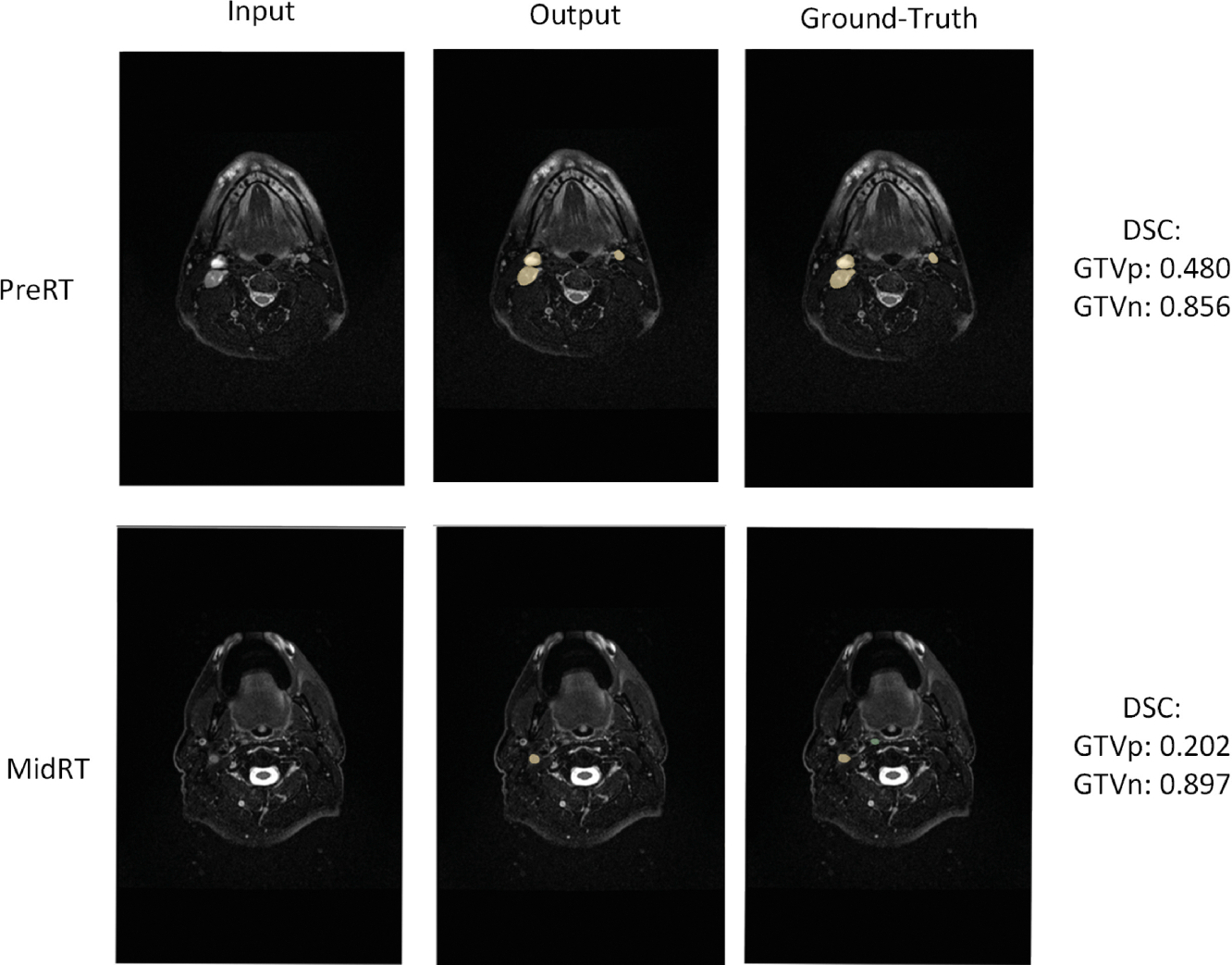
Exemplary visualization of the results for the preRT and midRT segmentation tasks (GTVp in green, GTVn in yellow). (Color figure online)

We follow the convention of the challenge organizers and use the aggregated Dice score (DSCAgg) to compare the architectures. The results using an internal 5-fold cross-validation are presented in Table 1. Based on the internal evaluation, we decided to use the RUNet as the final architecture and evaluated it on the closed test set. The final results for both the preRT and midRT subtasks are presented in Table 2. The results of our experiments performed well in comparison to prior contributions (DSCAgg at the best-performing submission level in the HECKTOR Challenge dedicated to HN cancer segmentation in PET-CT [1]), however, considerably worse than other methods based on nnUNet [10]. Figure 2 presents exemplary segmentation results with strong performance.

## 4 Discussion

The results confirm that the complexity of network architecture is not beneficial for the HN tumor segmentation, at least in low data regimes. The inductive bias in CNNs may be beneficial for tasks suffering from low amounts of data. The traditional RUNet outperforms more recent architectures like UNETR, SwinUNETR, or SegMamba. The most probable reason behind such an observation is connected with a relatively low amount of training data, limiting the expressiveness of transformer-based architectures. Therefore, in the final submission, we decided to use the RUNet, especially when it turned out to be impossible to combine RUNet and SwinUNETR models for external validation without reducing the number of parameters or volume resolution, due to the limitations of the GC hardware (T4 GPU with 16 GB of available VRAM). The models could not be loaded and process the volume simultaneously, and processing them independently with late aggregation would increase the inference time beyond the allowed limits.

Interestingly, the differences between the RUNet and transformer-based architectures are lower for midRT tasks. In terms of the GTVp, the SwinUNETR acquired even slightly better results (however, without statistically significant differences, p-value ¿ 0.05). The reason behind that is probably connected with the fact that we decided to use the three-channel input for the midRT tasks (the midRT volume, the registered preRT volume, and the registered preRT ground truth). By design, this helps the network to perform the initial detection and helps with the training convergence. Nevertheless, such an approach has also disadvantages. The network is unable to segment tumors that are not available (e.g. metastases) or were incorrectly segmented before the radiation therapy. However, such cases are outside the scope of the challenge. Due to the metrics used for the evaluation, we decided to use the three-channel input instead of using only the midRT volume. The decision is connected with the DSCAgg used for evaluation. The metric calculates the volumetric overlap using the entire test set. As a result, accurate segmentation of large segmentation masks is preferred over sensitivity to small lesions that barely impact the final metric. The correct detection of small changes in midRT volume is less influential than the accurate segmentation of the large tumors already visible in the associated preRT volume. For such cases, it would be more beneficial to use the average or median of individual Dice scores instead.

Importantly, we used only data provided by the challenge organizers. Probably external data used for pretraining the transformer-based architectures using self-supervised techniques (e.g. masked autoencoding) could improve the results. However, it would require to either use large-scale MRI datasets or to use the already existing foundational models dedicated to MR volumes [4]. The selfsupervised pretraining to each task separately would be suboptimal, it would require performing large-scale and computationally expensive initialization for each task separately. Therefore, a universal foundation model is necessary to make the approach useful. Current advances in deep learning suggest that this may be the most influential research direction to improve segmentation results [2].

To conclude, the paper presents a benchmark of several encoder-decoder architectures and suggests that in low data regimes, the more recent transformerbased architectures are not improving the segmentation results. Probably large-scale self-supervised pretraining could change the outcomes, however, to make it useful and scalable, such an approach would require proposing a universal foundation model dedicated to MR volumes. Therefore, the next research step would be related to developing or fine-tuning a large-scale foundation model dedicated to medical image segmentation.

## Figures and Tables

**Table 1.** Quantitative results for the internal evaluation using 5-fold cross-validation split based on aggregating results from 5 folds in terms of the aggregated Dice score (DSCAgg)

Model	GTVn ↑	GTVp ↑	Mean GTV ↑
Before Radiation Therapy (preRT)			
RUNet	**0.809**	**0.741**	**0.775**
UNETR	0.642	0.592	0.617
SwinUNETR	0.789	0.724	0.757
SegMamba	0.743	0.672	0.708
During Radiation Therapy (midRT)			
RUNet	**0.787**	0.614	**0.701**
UNETR	0.751	0.562	0.657
SwinUNETR	0.785	**0.616**	0.700
SegMamba	0.772	0.594	0.683

**Table 2.** Quantitative results on the closed test set using the Grand-Challenge evaluation platform in terms of the aggregated Dice score (DSCAgg) for both the subtasks (before and during the radiation therapy).

Task	GTVn ↑	GTVp ↑	Mean GTV ↑
preRT	0.826	0.717	0.771
midRT	0.836	0.579	0.707
